# Adhesion stimulates Scar/WAVE phosphorylation in mammalian cells

**DOI:** 10.1080/19420889.2020.1855854

**Published:** 2020-12-28

**Authors:** Shashi Prakash Singh, Robert H. Insall

**Affiliations:** a CRUK Beatson Institute, Glasgow, UK; b Institute of Cancer Sciences, University of Glasgow, Glasgow, UK

**Keywords:** Scar/WAVE, phosphorylation, cell migration, adhesion

## Abstract

The Scar/WAVE complex catalyzes the protrusion of pseudopods and lamellipods, and is therefore a principal regulator of cell migration. However, it is unclear how its activity is regulated, beyond a dependence on Rac. Phosphorylation of the proline-rich region, by kinases such as Erk2, has been suggested as an upstream activator. We have recently reported that phosphorylation is not required for complex activation. Rather, it occurs after Scar/WAVE has been activated, and acts as a modulator. Neither chemoattractant signaling nor Erk2 affects the amount of phosphorylation, though in Dictyostelium it is promoted by cell-substrate adhesion. We now report that cell-substrate adhesion also promotes Scar/WAVE2 phosphorylation in mammalian cells, suggesting that the process is evolutionarily conserved.

## Introduction

Actin polymerization at protrusions such as pseudopods is an essential part of cell movement. They are initiated by the Scar/WAVE complex, which drives the activation of Arp2/3 complex, leading in turn to the enrichment of actin in the leading edges, lamellipodia, or pseudopodia [1]. The conserved ~450 kDa complex is an assembly of five subunits, namely Pir121/CYFIP, Nap1/NckAP1, Scar/WAVE, Abi, and HSPC300/Brk [2].

One known regulator of Scar/WAVE is the small GTPase Rac1 [3]. When inactive Rac1 is GDP bound and in response to signaling cues Rac1-GDP is converted into Rac1-GTP and becomes active. The active form of Rac1 binds to the Pir121 subunit and is essential for the activation [4,5]. A number of other proteins also modulate the complex. CYRI locally inhibits activation of Scar/WAVE by buffering the GTP-bound Rac1 and control the dynamics of cell protrusions [6]. In contrast, Lamellipodin co-operates Scar/WAVE complex activation [7].

Phosphorylation of Scar/WAVE was described by several authors as a key activation mechanism; in most cases, this was before manipulation of the complex *in vivo* was tractable, so data about physiological roles of phosphorylation were lacking. Various domains of Scar/WAVE are phosphorylated at multiple positions. In the so-called “meander region”, tyrosine phosphorylation was found to control the activation of the complex [3]. Serine phosphorylation in the C-terminal VCA domain tunes the activity of the complex [8,9], though its activity appears to be negative – maintaining an inhibited closed state – rather than activating [9]. Similarly, older biochemical papers suggest that phosphorylation of Scar/WAVE’s proline-rich motif activates the complex [7,10–12]. However, we have now shown using gene replacements in knockout cells that in both Dictyostelium and cancer cells, phosphorylation in the proline-rich motif is neither a requirement for activation nor deactivation [13]. The phosphorylation only occurs following activation of the complex (in other words, it cannot be an upstream regulation) and tunes the activity of the complex. One interesting side observation is that this establishes Scar/WAVE phosphorylation as a readout of complex activation.

We also found that the upstream driver of Scar/WAVE phosphorylation was not the expected one – chemoattractant signaling, perhaps acting through Erk2, whose activity is strongly induced by signaling [14]. Instead, the principal correlates with phosphorylation of Scar in Dictyostelium was cell-substrate adhesion. When suspension-grown cells were allowed to adhere to glass, increased Scar phosphorylation was discernible at the earliest time points (2ʹ after contact), and deadhesion caused an immediate drop in phosphorylated bands. In this report, we have examined the phosphorylation of Scar homolog WAVE2 in mammalian cells that grow in suspension to cells that grow in adhesion. Cells that grow in adhesion have more WAVE2 phosphorylation compared with suspension cells.

## Materials and methods

### Cell culture

Jurkat and JVM3 Cells were grown in RPMI-1640 supplemented with 10% fetal bovine serum and 1% L-glutamine in tissue culture flasks at 37°C, 5% CO_2_. All other cell lines were grown in DMEM supplemented with 10% fetal bovine serum or donor bovine serum and 1% L-glutamine in tissue culture dishes at 37°C, 5% CO_2._

### Western blotting

Cells were washed twice with ice-cold PBS and lysed with RIPA buffer and kept on ice for 5 min. Cell debris was cleared by centrifugation at 13,000 rpm, 5 min, 4°C. Protein samples were boiled in 1X NuPAGE LDS buffer (Invitrogen) for 5 min and analyzed by western blotting. Proteins were resolved on self-made low-bis acrylamide (0.06% bis-acrylamide and 10% acrylamide) gels. Electrophoresis was performed at 150 V, 90 min. Proteins were electro-transferred onto nitrocellulose membrane using iblot (Invitrogen). Membranes were blocked in TBS+5% nonfat milk for 1 h at room temperature. Membranes were incubated overnight at 4°C with anti-WAVE2 (Cell signaling) and anti-tubulin (Sigma) at 1:1000 and 1:5000 dilution, respectively, in TBST+5% BSA. After washing with TBST three times for 5 min each, membranes were incubated with fluorescently conjugated secondary antibody (1:10,000 in TBST+5% BSA) at 4°C, 1 h. Membranes were washed three times with TBST and protein bands were detected by Odyssey CLx Imaging System (LI-COR Biosciences).

For phosphatase treatment, cells were lysed in TN/T buffer (10 mM Tris-HCl, pH7.5, 150 mM NaCl and 1% Triton X-100) and kept on ice for 5 min. Lysate was centrifuged (16,000 g, 4°C, 5 min) to remove the cell debris. To dephosphorylate the proteins, lysate was incubated at 30°C, 1 h with lambda phosphatase (NEB). Protein samples were boiled in 1X NuPAGE LDS buffer (Invitrogen) for 5 min and analyzed by western blotting.

### Quantification of protein bands

Protein band densities were calculated by ImageJ. A vertical line was drawn on the protein bands and intensity values of the top (phosphorylated) and bottom de-phosphorylated bands of WAVE2 were noted. The ratio of the top and bottom bands was used to represent the phosphorylation of WAVE 2 in the graph.

## Results and discussion

**Figure 1.**
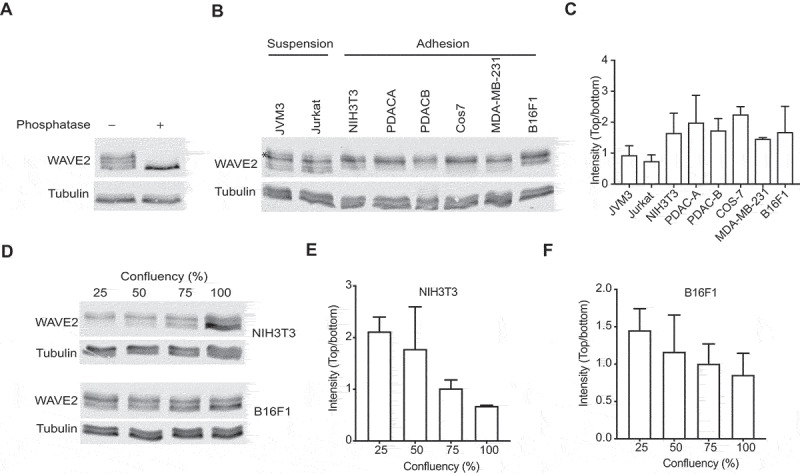
Adhesion stimulates Scar/WAVE2 phosphorylation. A)Western blot of B16F1 Scar/WAVE2 after phosphatase treatment. Whole cells were lysed in TN/T buffer and then incubated with lambda-phosphatase for 1 h at 30°C, boiled in sample buffer and analyzed using low-bis acrylamide gels. B) WAVE2 phosphorylation in mammalian cells. Lysates from cells that grow in suspension (Jurkat and JVM3) and adhesion (NIH3T3, PDAC-A, PDAC-B, Cos7, MDA-MB231, and B16F1) were analyzed for WAVE2 band shifts by western blotting. Suspension cells have additional lower bands (arrow) and less intense phosphorylated (*) band compared that grow in adhesion. C) Graph shows the ratio of the upper intense band (*) and the lowest band of various mammalian cells. (n = 3, mean ± SD). D) Effect of cell density on WAVE2 phosphorylation. NIH3T3 and B16F1 cells were grown at indicated cell densities and lysates were analyzed for WAVE2 band shifts. Western blotting shows that WAVE2 phosphorylation decreases as the cell density increases. * indicates phospho-Scar/WAVE. E) Graph shows the ratio of the upper intense band of WAVE2 in NIH3T3 cells as confluence increases (n = 3, mean ± SD, p = 0.01, Kruskal-Wallis test). F) Graph shows the ratio of the upper intense band of WAVE2 in B16F1 cells. (n = 3, mean ± SD, p = 0.4, Kruskal-Wallis test)

In our recent paper [13], we found that the physical adhesion of cells to the substratum stimulates Scar/WAVE phosphorylation. We previously only examined this phenomenon in Dictyostelium. To determine if Scar/WAVE2 phosphorylation in mammalian cells is also driven by cell:substratum adhesion, we examined western blots in which the PAGE separation used low-bis gels, which reveal different numbers of phosphorylations as positional shifts in the bands; more phosphates gives higher bands (Figure 1(a); 13). We compared the band shifts in Scar/WAVE2 from cells that preferentially grow in suspension (Jurkat and JVM3) with Scar/WAVE2 from cells that require adhesion to a surface (NIH3T3, the pancreatic cancer lines PDACA & B, Cos7, MDA-MB-231, and B16F1). The results, shown in Figure 1(b), reveal two changes. First, Jurkat and JVM3 cells have less intense upper bands, meaning highly phosphorylated (i.e., activated) Scar/WAVE2 is less abundant. This is consistent with the results from Dictyostelium cells, in which activation required adhesion [13]. Both suspension-grown cell types also reveal an additional band, representing less-phosphorylated Scar/WAVE2, that is barely discernible in cells that grown in adherent conditions. The intensity ratios of the upper intense and lowest bands present in all cells showed a marked increase in adherent cells compared with suspension cells (Figure 1(c)). We also modulated the amount of adhesion cells could achieve by varying cell density. Cells were grown at low to high density in tissue-culture dishes, and WAVE2 was analyzed for band shifts. Cells grown at high confluence have a lower adhesion area due to space constraints imposed by neighboring cells. The amount of Scar/WAVE2 phosphorylation decreases with confluence in both NIH3T3 and B16F1 cells (Figure 1d). Again, this is consistent with mammalian Scar/WAVE2 phosphorylation being stimulated by cell-substrate adhesion. Since we have shown this is a measure of its activation, the reduced Scar/WAVE2 phosphorylation implies that Scar/WAVE, in general, requires cell:substrate adhesion to be efficiently activated.

The connection between cell-substrate adhesion is novel but not hugely surprising. Many cell types require adhesion to a substrate to form pseudopods or lamellipodia [15]. Cell-substrate adhesion also leads to sharp relocalization of the Scar/WAVE complex [16]. However, one point remains a mystery. The connection between adhesion and Scar/WAVE activation seems to be mechanical. Talin knockouts, which show effectively zero cell:substrate adhesion, also show little Scar/WAVE phosphorylation. But when adhesion is forced artificially, by squeezing the cells with a layer of agarose, Scar/WAVE phosphorylation is restored, though talin and the pathways downstream are still blocked. Thus, some physical correlate of adhesion, rather than adhesion-based signaling, seems to be the key. We look forward to the future elucidation of how this can work.
